# Knockdown of MBP-1 in Human Foreskin Fibroblasts Induces p53-p21 Dependent Senescence

**DOI:** 10.1371/journal.pone.0003384

**Published:** 2008-10-13

**Authors:** Asish K. Ghosh, Tatsuo Kanda, Robert Steele, Ratna B. Ray

**Affiliations:** 1 Department of Pathology, Saint Louis University, St. Louis, Missouri, United States of America; 2 Cancer Center, Saint Louis University, St. Louis, Missouri, United States of America; Ordway Research Institute, United States of America

## Abstract

MBP-1 acts as a general transcriptional repressor. Overexpression of MBP-1 induces cell death in a number of cancer cells and regresses tumor growth. However, the function of endogenous MBP-1 in normal cell growth regulation remains unknown. To unravel the role of endogenous MBP-1, we knocked down MBP-1 expression in primary human foreskin fibroblasts (HFF) by RNA interference. Knockdown of MBP-1 in HFF (HFF-MBPsi-4) resulted in an induction of premature senescence, displayed flattened cell morphology, and increased senescence-associated beta-galactosidase activity. FACS analysis of HFF-MBPsi-4 revealed accumulation of a high number of cells in the G1-phase. A significant upregulation of cyclin D1 and reduction of cyclin A was detected in HFF-MBPsi-4 as compared to control HFF. Senescent fibroblasts exhibited enhanced expression of phosphorylated and acetylated p53, and cyclin-dependent kinase inhibitor, p21. Further analysis suggested that promyolocytic leukemia protein (PML) bodies are dramatically increased in HFF-MBPsi-4. Together, these results demonstrated that knockdown of endogenous MBP-1 is involved in cellular senescence of HFF through p53-p21 pathway.

## Introduction

MBP-1, an ∼37 kDa cellular protein, has multiple functions. It binds to the c-myc promoter sequences and transcriptionally represses the c-myc gene. MBP-1 acts as a general transcriptional repressor [1]–[3]. Sequence analysis suggested that MBP-1 has a high homology with ENO1 cDNA, an ∼48 kDa protein, designated as human enolase cDNA [1], [4]. However, the enolase enzymatic activity was not demonstrated from this ENO1 cDNA clone. Whether full length ENO1 gene product has a similar function like MBP-1 in carcinoma cells is yet to be determined. However, most of the studies to date used MBP-1 cDNA which expresses ∼37 kDa protein. Structure/function analysis of MBP-1 mutants revealed that the transcriptional repressor domains are located in the amino-terminal (MBP-AR) and carboxy-terminal (MBP-CR) regions. We have demonstrated that MBP-1 exerts an anti-proliferative effect on a number of cancer cell lines and inhibits tumor growth in nude mice [5], [6]. While the role of exogenous expression of MBP-1 in the transcription and cell growth regulation appear to be established, the *in vivo* function of this protein is poorly understood.

Normal human cells respond to certain types of DNA damage caused by histone deacetylase inhibitors (which remodel chromatin) and oncogenic forms of Ras or Raf (which transduce mitogenic signals) by adopting a phenotype that closely resembles replicative senescence [7]. On the other hand, immortalized cells tend to respond to DNA damage or oncogenes by undergoing apoptosis or neoplastic transformation. Cell senescence is defined as proliferative arrest that occurs in normal cells after a limited number of cell division. Cells that underwent senescence cannot divide even if stimulated by mitogens, but they remain metabolically active and show characteristic changes in morphology, such as enlarged and flattened cell shape and increased granularity [8]. Senescence is controlled by two major tumor suppressors, the p53 gene and the retinoblastoma (Rb) gene [9]–[11]. An increase in p53 transcriptional activity is a molecular signature for cellular senescence. The increased activity is driven by changes in p53 phosphorylation and acetylation status [12], [13]. The senescent-associated growth arrest is due to the downregulation of selected positive-acting cell cycle regulatory genes. The activities of cyclin-dependent kinase 2 (Cdk2) and cyclin-dependent kinase 4 (Cdk4) are greatly reduced, due to the increased expression of the Cdk inhibitor proteins p21, and p16, causing Rb to be present in its hypophosphorylated form.

In this study, we have uncovered a novel function of endogenous MBP-1. Knockdown of MBP-1 in human primary fibroblasts induced premature senescence involving the p53-p21 signaling pathway.

## Results

### Knockdown of endogenous MBP-1 in human foreskin fibroblasts results in decreased cell proliferation

**Figure 1:**
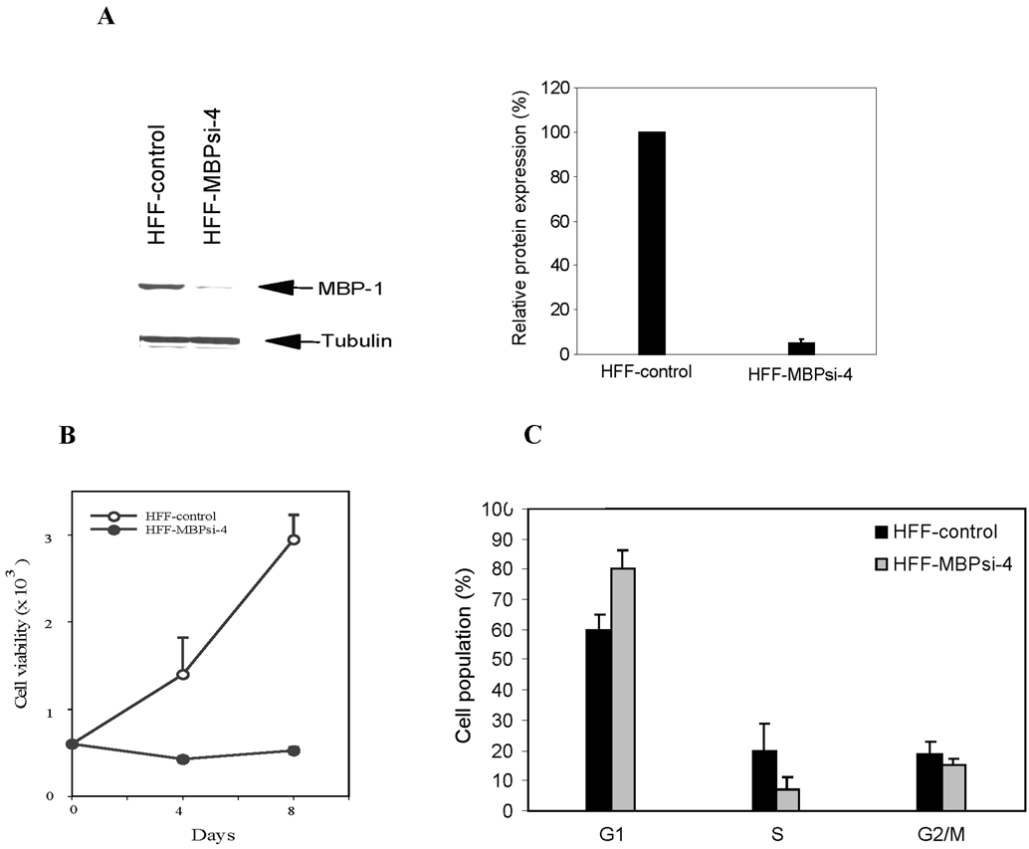
Knockdown of MBP-1 in human foreskin fibroblasts induces cell proliferation. Panel A: Normal human foreskin fibroblasts were transfected with MBP-1 specific shRNA or control scrambled shRNA and selected with neomycin for 3 weeks. Colonies were pooled and examined for endogenous expression of MBP-1 by Western blot analysis using a specific antibody to MBP-1. Densitometric analysis of MBP-1/tubulin was performed using Image Quant Software (Amersham, ME). Panel B: Knockdown of MBP-1 inhibits HFF proliferation. Cells were plated after selection, and counted at different time points by trypan blue exclusion method. Panel C: MBP-1 knockdown in HFF results in accumulation of HFF at the G1 phase. Asynchronized HFF-control and HFF-MBPsi-4 were harvested fixed and stained with propidium iodide. DNA content was analyzed by flow cytometry. Results are represented as percent of cell population in G1, S, and G2/M phases of the cell cycle. Results are presented together with standard errors from three independent experiments.

To investigate the role of endogenous MBP-1 in cellular proliferation, we knocked down endogenous MBP-1 in human foreskin fibroblasts (HFF) using RNA interference. Initially we have a used several siRNAs, and two of them efficiently knockdown MBP-1 expression [14]. For generation of stable clone, we constructed a plasmid DNA vector expressing a potent shRNA targeted to MBP-1 coding region or scrambled shRNA. HFFs were transfected with the plasmid DNA pRNAH1.1-MBPsi-4 (HFF-MBPsi-4) or scrambled shRNA (HFF-control), selected for neomycin resistant colonies and pooled to avoid clonal selection. Cell lysates were prepared for Western blot analysis to detect endogenous expression of MBP-1 using a specific antibody. We observed 95% inhibition of MBP-1 in HFF-MBPsi-4 as compared with that of HFF-control (Fig. 1, panel A). Similar results were obtained using MBPsi-3, suggesting that observed effect is not off-target. We have also used three different pools of transfectants and observed similar results. For subsequence studies, we have utilized HFF-MBPsi-4. We examined whether knockdown of MBP-1 has an effect on cell proliferation. For this, cell proliferation of MBP-1 knockdown HFF was determined after antibiotic selection. HFF-MBPsi-4 exhibited a significantly slower rate of proliferation as compared with the HFF-control suggesting that depletion of MBP-1 inhibited cell proliferation (Fig. 1, panel B). Senescent cells remained viable for 8 weeks, and then slowly disintegrated. Human primary fibroblasts generally display senescence after 70–80 passages. Next, we examined whether knockdown of MBP-1 modulates cell cycle progression in HFF-MBPsi-4 by FACS analysis. An equal number of HFF or HFF-MBPsi-4 (10^5^ cells) were seeded and grown for 24 h. Cells were then trypsinized and stained with propidium iodide for FACS analysis (Fig. 1, panel C). An approximately 80% of HFF-MBPsi-4 cells accumulated in G1 phase as compared to ∼60% of control fibroblasts. Cells accumulated in the G2/M phase did not exhibit a significant difference between the control and HFF-MBPsi-4. However, we observed a ∼3-fold reduction in the number of HFF-MBPsi-4 accumulated in the S-phase of the cell cycle as compared to that of the control cells. Together, our results suggested that MBP-1 knockdown interrupts progression of the cell cycle from the G1 to S phase in normal HFFs.

### Inhibition of MBP-1 in HFF induces premature senescence

**Figure 2:**
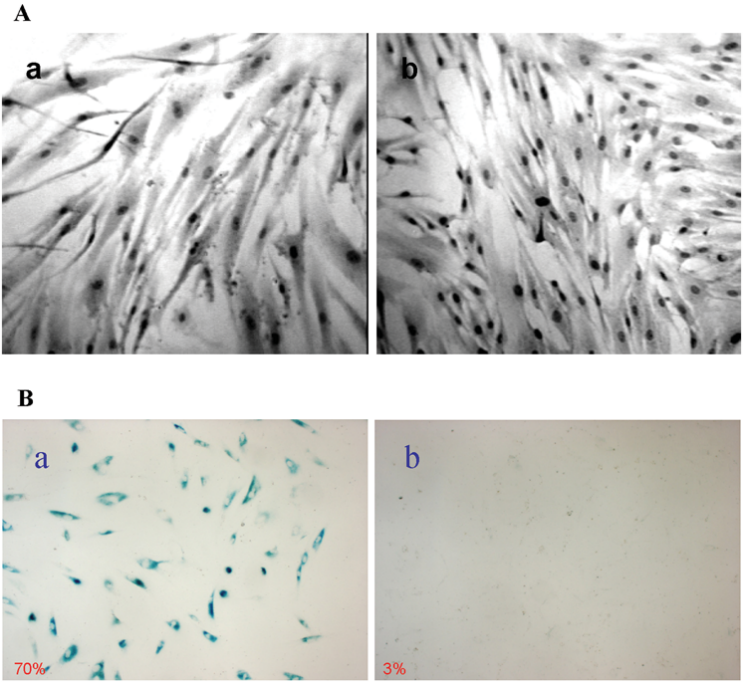
Knockdown of MBP-1 in HFF altered morphology and exhibited senescence-associated β-galactosidase expression. Panel A: HFF-MBPsi-4 (panel a) and HFF-control (panel b) were grown to near confluency and stained with H & E. MBP-1 knockdown fibroblasts displayed an irregular, enlarged cellular morphology as compared to the control siRNA transfected cells. Panel B: HFF-MBPsi-4 cells and control-HFF were grown in plates and senescence-associated β-galactosidase assay was performed. MBP-1 knockdown HFF displayed a significantly higher senescence associated β-galactosidase activity (panel a), unlike in control HFF (panel b).

During the period of monitoring cell viability, we observed that HFF-MBPsi-4 was growing larger in size. To ascertain our observation, we plated an equal number of control HFF and HFF-MBPsi-4. Cells were grown to confluency and stained with hematoxylin and eosin (H & E). HFF-MBPsi-4 diaplayed increased in size as compared to HFF-control cells (Fig. 2, panel A) using light microscopy under same magnification. We have also observed that MBP-1 knocked down HFFs displayed a flattend cellular morphology with progressive cell passage. To investigate whether MBP-1 knockdown leads to growth arrest, HFF-control or HFF-MBPsi-4 were plated for senescence associated β-galactosidase assay (SA-β-gal). Detection of β-galactosidase activity at pH 6.0 is a known characteristic of senescent cells. After 48 h, cells were fixed and stained with X-gal for the detection of β-galactosidase activity (Fig. 2, panel B). More than 70% of HFF-MBPsi-4 displayed appearance of enlarged blue cells (panel a) in contrast to the control HFF cells, which failed to exhibit a detectable blue appearance (panel b). Therefore, these results suggested that knockdown of MBP-1 leads HFFs to undergo premature senescence.

### Knockdown of MBP-1 in HFF is associated with the modulation of cyclins

**Figure 3:**
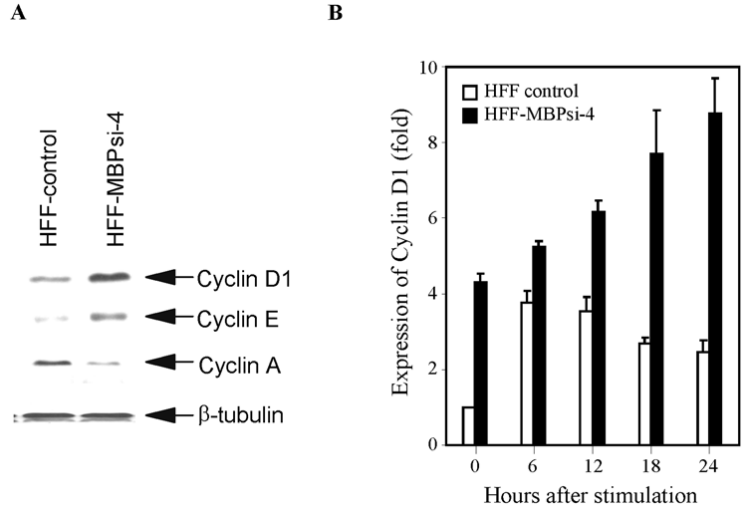
MBP-1 knockdown in HFF modulates expression of cyclins. Panel A: Expression of cyclins in control and MBP-1 knockdown HFF. Lysates prepared from cells were subjected to Western blot analysis using indicated antibodies. Knockdown of MBP-1 enhanced cyclin D1 expression and cyclin E and reduced cyclin A expression in HFF-control. Panel B: Time-dependent elevation of cyclin D1 expression in MBP-1 knockdown HFF as compared to control HFF. Cells were synchronized by serum-starvation and lysates were prepared at different time points as indicated following serum stimulation. Cell lysates were examined for the expression of cyclin D1 by Western blot analysis using specific antibody. Protein load was normalized and determined level of cyclin D1 by densitometric analysis. Arbitrary unit was chosen to represent relative cyclin D1 expression. Results are presented together with standard errors from three independent experiments.

HFF-MBPsi-4 and HFF-control lysates were analyzed for the expression of cyclin D1, cyclin E, and cyclin A using specific antibodies. We have observed that cyclin D1 and cyclin E expression was significantly upregulated (4 and 2 fold, respectively) in HFF-MBPsi-4 as compared to HFF-control (Fig. 3, panel A). However, cyclin A expression was markedly lower (∼3 fold) in MBP-1 knockdown HFF. Next, HFF-MBPsi-4 and HFF- control were synchronized by serum starvation and cell lysates were prepared at 0, 6, 12, 18, and 24 h following serum stimulation. The lysates were analyzed for the expression of cyclin D1. The basal level (0 h) of cyclin D1 in control HFF was significantly lower (∼4-fold) than the HFF-MBPsi-4. Elevated levels of cyclin D1 were detected in the HFF-control between 6–12 h which decreases afterwards. In contrast, the level of cyclin D1 expression in HFF-MBPsi-4 was increasing with time (Fig. 3, panel B). Senescent human cells generally arrest growth with a G_1_ DNA content, and cannot be stimulated to divide by physiological mitogens. Higher expression of cyclin D1 in HFF-MBPsi-4 further suggested the permanent growth arrest in HFF upon knockdown of MBP-1.

### Post-translational modification of p53 occurs in MBP-1 knockdown HFF

**Figure 4:**
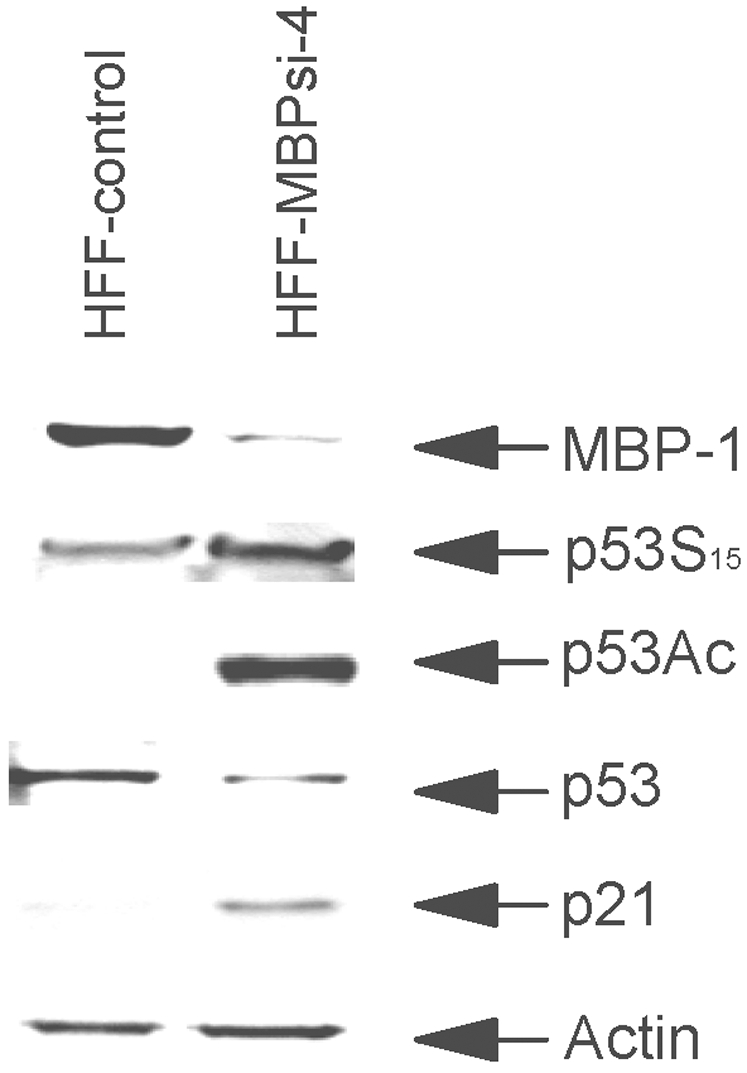
MBP-1 knockdown results in activation of p53 and p21. Lysates from HFF-control and HFF-MBPsi-4 were subjected to Western blot analysis by probing with antibodies against MBP-1, phosphorylated (Ser15), acetylated p53(Lys382), p53 (DO1), p21 and actin.

An increase in p53 transcriptional activity is a molecular signature for cellular senescence. The increased activity is driven by changes in p53 phosphorylation (at Ser15) and acetylation (at Lys382) status [12], [13]. To investigate whether pathway linking p53 and senescence after knocking down MBP-1 in HFFs, we examined the post-translational modification of p53 by Western blot analysis using specific antibodies. We have observed p53 acetylation at Lys382 site and phosphorylation at Ser15 in MBP-1 knockdown HFF (Fig. 4). These results suggest that MBP-1 knockdown in HFF results in activation of p53. Enhancement of p53 by phosphorylation and/or acetylation results in activation of its target genes. The cyclin dependent kinase inhibitor, p21 is one of the p53 target genes. Therefore, we examined whether an increase in p21 expression in HFF-MBPsi-4 was due to activation of p53 transcriptional activity. Our results demonstrated a significant increase in p21 in the HFF-MBPsi-4 as compared to HFF-control.

### Cell cycle regulatory genes are modulated in HFF-MBPsi-4

To gain mechanistic insights into MBP-1 for cellular functions, we used a pathway specific gene array to identify MBP-1 target genes in MBP-1 knockdown HFF by comparison with controls. We isolated total RNAs from HFF-control and HFF-MBPsi-4 for studying the influence of endogenous MBP-1 on cell cycle gene expression using a cell-cycle pathway specific mini array. Growth inhibition was indicated by an induction of p53 target genes, p21 and GADD45A, and decreased expression of genes driving progression through various phases of the cell cycle. Notably, several genes encoding critical components of the DNA replication machinery (MCM4/MCM5) were down-regulated. A representative subset of the results is presented in Table 1. Interestingly, we have observed downregulation of the p27/KIP1 gene in senescent HFF. On the other hand, Collado et al. [15] have shown that premature senescence mediated by LY298004 in MEFs display accumulation of the p27. The difference of these results could be due to a difference between human vs. mouse cells or mediator selectively targeting p21 or p27 gene. Lysates from the HFF-control or HFF-MBPsi-4 were analyzed for expression of p21 and cyclin D1 by Western blot using specific antibodies. A significant elevation in p21 and cyclin D1 expression was observed in the lysates of HFF-MBPsi-4 when compared with control cells (Figs. 3 and 4), which corroborates with our gene array data.

**Table 1:** Genes differentially expressed in MBP-1 depleted HFF.

Biological Process	Gene Name	Fold Change	Gene Title
**G1 Phase and G1/S Transition**	CCND1	7.46	Cyclin D1
	CDK6	4.04	Cyclin-dependent kinase 6
**DNA Replication**	MCM4	−3.27	minichromosome maintenance deficient 4
	MCM5	−3.2	minichromosome maintenance deficient 5
**Cell Cycle Checkpoint and Arrest**	GADD45A	5.35	growth arrest and DNA-damage-inducible 45 alpha
	CDKN1A	4.77	cyclin-dependent kinase inhibitor 1A (p21)
	BRCA2	−3.4	breast cancer 2
	CEDKN3	−5.45	cyclin-dependent kinase inhibitor
**Regulation of Cell Cycle**	CDKN1B	−3.56	cyclin-dependent kinase inhibitor 1B (p27)
	CCNB1	−3.63	cyclin B1
	CCNB2	−4.61	cyclin B2
	CDC2	−4.77	cell division cycle 2 homolog
	CDC20	−6.57	cell division cycle 20 homolog
	RAD9A	−7.06	RAD9 homolog
	MKI67	−7.67	antigen identified by monoclonal antibody Ki 67
**Negative Regulation of Cell Cycle**	RBL1	−3.08	retinoblastoma-like 1 (p107)

### MBP-1 knockdown in HFF induces accumulation of PODs

**Figure 5:**
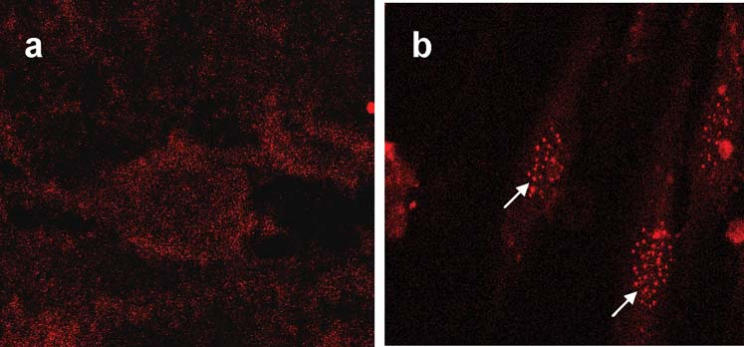
PODs accumulate during premature senescence in MBP-1 knockdown HFF. PML localization in HFF-control (Panel A) or HFF-MBPsi-4 (Panel B) was determined by indirect immunofluorescence using the PML monoclonal antibody.

PML plays a critical role in growth control, transformation suppression, induction of apoptosis, and replicative senescence depending on the cellular context. PML is upregulated during cellular senescence, and induces premature senescence in response to oncogenic Ras by promoting p53 acetylation [16]. PML is the defining component of nuclear structures known as promyelocytic oncogenic domains (PODs). The ability of PML to PODs appears essential for its prosenescence activity. The accumulation of PODs in response to oncogenic Ras is coincident with the onset of premature senescence and appears unique to the senescent state. To determine whether PML is induced in MBP-1 knockdown HFF, we conducted immunofluorescence microscopy on HFF-control and HFF-MBPsi-4 using a PML monoclonal antibody. We have observed that the PML nuclear bodies increase in size and number in HFF-MBPsi-4 as compared to HFF-control (Fig. 5), and may play a pivotal role in the senescence process.

## Discussion

The normal cellular function of endogenous MBP-1 remains unclear. To investigate the endogenous function, we knocked down MBP-1 expression in HFF by RNA interference. Knockdown of MBP-1 expression in HFF (>95%) resulted in significant inhibition of cell proliferation as compared to parental control cells. Morphological studies after knockdown of MBP-1 revealed enlarged and flattened cell morphology and positive senescence-associated β-galactosidase staining, characteristics of senescent fibroblasts. Cell cycle analysis demonstrated an accumulation of ∼80% MBP-1 knockdown cells in the G1-phase as compared to ∼60% control fibroblasts. Although no significant difference was observed in G2/M phase, only ∼7% of MBP-1 knockdown HFF accumulated in the S-phase as compared to control HFF (20%). To gain mechanistic insights, we examined the expression level of different cyclins. A high level expression of cyclin D1 (∼4 fold) and cyclin E (∼2 fold) was detected in MBP-1 knockdown HFF as compared to the control fibroblasts. On the other hand, expression of cyclin A, one of the target genes of Rb/E2F1 signaling pathway, was significantly lower (∼3 fold) in the senescent fibroblasts.

MBP-1 knocked down fibroblasts displayed enhanced phosphorylation and acetylation of p53 suggesting an upregulation of p53 transcriptional activity. The activity of p53 is regulated by recruitment into PML-nuclear bodies as well as stabilization through post-translational modifications, such as phosphorylation and acetylation. PML nuclear bodies are also implicated in diverse cellular functions, such as gene regulation, apoptosis, and senescence. Knockdown of MBP-1 in HFF increased PML accumulation in the nucleus, which may enhance PML-induced acetylation of p53 and PML-induced cellular senescence, although future study is necessary in understanding the detailed mechanism. Inhibition of superoxide dismutase (SOD) by RNAi induces premature senescence in normal human fibroblasts [17]. The senescent fibroblasts displayed elevated expression levels of p53 in the nucleus. Depletion of DNA methyltransferase in normal diploid human fibroblasts results in senescent-like growth arrest and is accompanied by up-regulation of p21 [17]. We have also observed that the senescent fibroblasts displayed a higher level p21 expression which may inhibit the kinase activities of cyclin D1-Cdk4/6 and cyclin E-Cdk2 complexes resulting in inhibition of cell cycle progression. We have observed a significant downregulation of wild type p21 promoter activity by MBP-1 in HFF as compared to p53 site mutated p21 promoter in an *in vitro* reporter assay. Cyclin D1-Cdk4/6 specific phosphorylation of Rb at Ser780 was only barely detectable in MBP-1 knockdown HFF, however p16 was not upregulated (data not shown).

The target genes controlled by endogenous MBP-1 remains unknown at present. Our results demonstrated that MBP-1 might regulate these genes through p53. The gene array analysis from MBP-1 knockdown HFF vs. control HFF yielded an expression signature that was indeed consistent with the observed phenotype for MBP-1 knockdown HFF, thereby imparting credibility to our data. Growth inhibition was indicated by an induction of p53 target gene p21 and decreased expression of genes driving progression through phases of the cell cycle, such as CDC2 and CDC20. Notably, several genes encoding critical components of DNA replication machinery were downregulated. Taken together these results indicated a role of endogenous MBP-1 in cell survival.

Opposing roles in cell growth were also observed with several genes, such as KLF4, E2F, VHL, Ras, p75 neurotrophin receptor (p75^NTR^), Maf, and TGF-β [19], [20]. Affar *et al* [21] recently reported that a reduction of YY1 level impairs embryonic growth and viability in a dose-dependent manner. Inactivation of tumor suppressor gene BRAC1 was also found embryonically lethal [22]. The HMGA proteins can promote tumorigenesis [23], although HMGA proteins have recently been shown to specifically accumulate in senescent cell chromatin, and contribute to the stable repression of proliferation-associated genes [24]. We have demonstrated previously that forced expression of MBP-1 negatively regulates cell growth in a number of human cancer cell lines of various tissue origins [6]. How might these contradictory activities of MBP-1 be explained? One possibility stems from the finding that overexpression of MBP-1 in cancer cells induces cell death, which suppresses tumorigenesis by inhibiting anti-apoptotic proteins [14], [25]. Here, we have shown that knockdown of MBP-1 induces premature senescence of primary human fibroblasts. Two connected yet distinct senescence pathways have been identified: one consisting of p53-p21 and the second p16-Rb [26]. Although RasV12-induced senescence involves both pathways [27], cellular senescence induced by the knockdown of MBP-1 involved only p53-p21. Knockdown of MBP-1 in human urothelial cells immortalized by HPV E6 did not exhibit senescence or upregulation of p21 (data not shown). However, it remains unclear which upstream stimuli preferentially use the p53-p21 pathway to trigger senescence. Identification of other MBP-1 interacting proteins to understand how these proteins interact with each other in the complex, and to find out exactly how these proteins interplay with p53 and repress its transcriptional activity will help to get insights into the normal physiological function of MBP-1.

## Materials and Methods

### Cell culture

Human foreskin fibroblasts (HFF) were grown in DMEM containing 10% fetal bovine serum (FBS) with penicillin/streptomycin in a 5%-CO_2_ incubator.

### Generation of HFF stably expressing MBP-1-specific shRNA

We have identified two potent siRNA sequences targeted to MBP-1 mRNA [14]. Both the siRNAs targeted to MBP-1 efficiently knockdown MBP-1 expression. We next cloned small hairpin RNAs (shRNAs) targeting MBP-1 (MBPsi-4) mRNA into a pRNAH1.1/neo plasmid vector (Genscript) under the control of the H1 promoter. Control scrambled sequences were used similarly. Early passage HFF (passage 4) were transfected with a plasmid DNA expressing MBP-1 specific (HFF-MBPsi-4) or control shRNA (HFF-control). After 48 h of transfection, cells were treated with 400 µg/ml G418 for selection of antibiotic-resistant colonies over a period of 3 weeks. All colonies were pooled and examined for the expression of endogenous MBP-1 by Western blot analysis using specific antibody. After selection, cell growth was measured.

### Senescence associated β-galactosidase assay

Equal number of HFF-control and HFF-MBPsi-4 were plated in 35 mm plates for the senescence associated β-galactosidase assay, as described earlier [28]. After 48 h, cells were fixed and stained with X-gal for detection of β-galactosidase activity using a senescence β-galactosidase staining kit (Cell Signaling). Cells exhibiting positive β-galatosidase activity (turned blue) at pH 6.0 were counted under a light microscope.

### FACS analysis and Western blot analysis

Cells were plated in DMEM containing 10% FBS and grown overnight. After 24 h, cells were trypsinized and fixed in ice-cold 70% ethanol overnight at 4°C. Cells were washed, stained with propidium iodide for 2 h and subjected to FACS analysis on a FACScan flow cytometer (BD PharMingen). Data were analyzed using the CellQuest and ModFit software. For the analysis of cell cycle regulatory cyclins, cells were serum starved (0.5%) and stimulated by adding complete DMEM containing 10% FBS. Cells were lysed in 2× SDS-sample buffer following serum stimulation at 0, 6, 12, 18, and 24 h. Cell lysates were then subjected to Western blot analyses using specific antibodies to various cyclins. Cell lysates were also prepared from unsynchronized HFF-control and HFF-MBPsi-4 and analyzed for MBP-1 expression, cyclin-dependent kinase inhibitor (p21), p53, phosphorylated and acetylated p53 by Western blot using specific antibodies. All the antibodies to cyclin D1, cyclin E, cyclin A and cyclin B1, p21 and p53 were purchased from Santa Cruz Biotechnology, acetylated p53 (Lys-373/382) from Upstate Biotechnology, phosphorylated p53 (Ser-15) and phospho-Rb (Ser-780) from Cell Signaling.

### Gene array

We isolated total RNAs from control and MBP-1 knockdown fibroblast for studying the influence of endogenous MBP-1 on cell cycle gene expression. Five µg of total RNA was used to produce cDNA using a First Strand cDNA Synthesis Kit (SuperArray). Quantitative amplification of the fibroblasts cDNAs was monitored with SYBR Green using real-time PCR. Amplification was carried out in 25 µl of ROX PCR Master Mix (SuperArray) containing 0.2 µM of each primer and 1 µl of the reverse transcription reaction mixture to each well of 96 well cell cycle array plate in a 7700 Real-time PCR system (Applied Biosystems) following the manufacturer's protocol. Data analysis was based on the ΔΔCt method with normalization of the target genes in MBP-1 knockdown fibroblasts to control fibroblasts. Data was analyzed using PCR Array data analysis software (SuperArray).

### Fluorescence Microscopy

HFF-control and HFF-MBPsi-4 were fixed in 3.5% paraformaldehyde for 15 min, permeabilized in 0.2% Triton X-100-PBS for 5 min, and blocked in 5% BSA in PBS at room temperature for 1 h. Primary antibody (anti-PML, PG-M3, 1∶200, Santa Cruz) incubation were performed for 1 h at room temperature. After washing the cells, secondary antibody incubation was performed with goat anti-mouse immunoglobulin G (IgG)-Alexa Fluor-546 for 45 min at room temperature. Control experiments demonstrated no detectable staining by secondary antibodies only (data not shown). Cells were visualized with a Nikon immunofluoroscence microscope.
